# Coercive measures in psychiatry: consensus and controversy in Swiss professional discourse

**DOI:** 10.3389/fpsyt.2026.1853183

**Published:** 2026-05-29

**Authors:** Matthias Jaeger, Anastasia Theodoridou

**Affiliations:** 1 Psychiatrie Baselland, Liestal, Switzerland; 2 University Hospital of Psychiatry, Zurich, Switzerland

**Keywords:** autonomy, discourse, ethics, prevention, Switzerland

## Abstract

**Introduction:**

Coercive measures, such as involuntary admission, treatment without consent and mechanical restraint, are among the most ethically contentious practices in psychiatry. In Switzerland, several influential position papers and guidelines articulating different professional, ethical and policy perspectives on the use and prevention of coercion have been published in recent years.

**Objective:**

This review aims to provide a comparative analysis of five key Swiss documents addressing coercive measures in psychiatry, focusing on their formal characteristics, thematic priorities and underlying normative assumptions.

**Methods:**

A narrative review was conducted aiming to synthesize ethical, legal and clinical perspectives on coercive measures in psychiatry, with particular focus on Swiss position papers emphasizing legal and ethical frameworks. The review included documents discussing involuntary admission, forced medication, seclusion, and restraint, as well as broader ethical analyses of coercion. Five documents published between 2014 and 2025 by various professional and stakeholder organizations were analyzed in terms of their formal structure, key recommendations and framing of coercion.

**Results:**

A broad consensus exists across all documents regarding the ethical problem of coercion, the importance of prevention and the prioritization of voluntary treatment. However, significant differences emerged with respect to normative goals (reduction vs. abolition of coercion), attribution of responsibility (procedural safeguards vs. relational practice vs. systemic reform), interpretation of clinical risk and security logics, and framing of coercion as exceptional necessity, professional failure or systemic symptom.

**Conclusions:**

The current Swiss debate among professionals on coercive measures is characterized less by empirical disagreement than by different normative and discursive rationalities. Future developments may benefit from integrating procedural and legal safeguards with preventive, relational, outcome-oriented and systemic approaches aimed at reducing coercion.

## 1 Introduction

Coercive measures in medicine, including involuntary admission, restricting movement, and treatment without consent, are among the most controversial interventions in medicine from professional, ethical and social perspectives (1). These measures touch upon fundamental rights, particularly personal freedom and bodily integrity, and place healthcare providers in the tension between autonomy, the duty of care and harm prevention (including public safety). Furthermore, they must be distinguished from those used in Forensics (a special form of medicine and a subspeciality of general psychiatry involving legal contexts such as criminal, correctional and regulatory matters) and from coercive/security measures regulated by police law (which have no connection to medicine).

Ethical considerations, such as those set out in Beauchamp and Childress’s principles-based approach, are widely recognized in psychiatric practice (2). The four principles of autonomy, beneficence, non-maleficence and justice are weighed against one another in each individual case. In this context, the debate regarding the balance between the protection of the individual and the protection of society takes on particular significance. Despite ongoing nuanced and critical reflection on this issue, coercion has been a feature of psychiatry since its establishment as a medical discipline. A recently published European study has demonstrated that coercive measures continue to exist or have even increased, despite political efforts to curb them (3).

In Switzerland, the revision of the Civil Code (https://www.fedlex.admin.ch/eli/cc/24/233_245_233/de) - Child and Adult Protection Act (2013) - led to a certain degree of standardization of legal principles (e.g. the right to appeal) at federal level, while allowing for differences in implementation at cantonal level (4). Data from the Federal Statistical Office and the National Quality Monitoring in Healthcare (Switzerland) showed that the absolute number of people admitted to inpatient psychiatric care against their will rose by 34.2% over a seven-year period (2016–2023), whilst the absolute number of psychiatric patients on whom coercive measures were applied increased by 55.8% over the same period (3). In 1997, the Swiss Medical Association (FMH) issued a code of conduct that regulates many principles of medical practice (5). This code contains rules of professional ethics pertaining particularly to the conduct of physicians in public, towards patients, towards colleagues and towards insurances. In practice, these rules strongly influence case law and legislation.

At the same time, there is a historically developed professional and societal debate that is currently reflected, among other things, in several position papers and guidelines. While these documents claim to provide normative guidance, they differ significantly, in their objectives, line of reasoning and implicit view of humanity. The five documents are position papers, recommendations, or demands issued by psychiatric professional associations, which are described in the respective sections. These are not legally binding or binding in any other way. Only the SAMW issues medical guidelines with a higher degree of binding force, which is also described in the following section. The five documents relevant to this review are presented below in terms of their structure and content.

### 1.1 Medical-ethical guidelines, “Coercive Measures in Medicine”, of the Swiss Academy of Medical Sciences

The SAMW (a private law foundation) is part of the Swiss Academies of Arts and Sciences. This non-profit association (www.samw.ch/de) brings together the private research funding institutions listed in the Federal Act on the Promotion of Research and Innovation. Since its foundation, the SAMW has primarily focused on the medical sciences in accordance with its founding purpose and it is actively involved in professional, policy and organizational matters. The SAMW’s guidelines are considered in case law by both cantonal courts and the Federal Supreme Court. The Swiss Federal Supreme Court treats the SAMW guidelines with great respect in its decisions and recognizes them, particularly in areas where state regulations are lacking or vague, as a relevant source of medical-ethical regulations of high authority. The first edition of the guidelines “Coercive Measures in Medicine” was published in 2015 (6). The guidelines form part of the FMH (Swiss Medical Association) Code of Ethics and are recommended for use by the Swiss Association of Nurses. The Central Ethics Committee of the SAMW has tasked a subcommittee composed of experts from various professional fields (medicine, nursing, law, ethics) with drafting medical-ethical guidelines on the topic of “Coercive Measures in Medicine.” The SAMW Senate has approved a first draft of these guidelines for consultation with professional societies, organizations, and interested individuals. The comments received have been incorporated into the final version. The definitive version of these guidelines was approved by the SAMW Senate on November 19, 2015. The document comprises 28 printed pages plus an appendix and is divided into four chapters: Scope of the guidelines, basic concepts and legal framework, principles, and fields of application. The appendix contains a multidimensional concept of coercion, guidance on how to implement the guidelines and a glossary. The chapter on principles essentially states that coercive measures are only permissible as a last resort when serious danger cannot be averted by other means. Such measures must be proportionate, appropriate, and necessary, and serve to protect the affected person or third parties. A prerequisite is a careful assessment of mental capacity and consideration of the presumed will or existing advance directives. Every coercive measure must be transparently justified, supported by an interdisciplinary team, limited in duration and fully documented. Furthermore, the dignity, rights and integrity of the affected person must be upheld at all times, and there is an obligation to conduct a debriefing and institutional quality assurance (6).

### 1.2 Recommendations for the prevention of restrictive measures in psychiatry by the Swiss Society of Psychiatry and Psychotherapy

The predecessor organization of the SGPP was founded in 1849. As the central professional association for psychiatry in Switzerland, its objectives include professional development, quality assurance, continuing education and training, and professional policy (www.psychiatrie.ch/sgpp). The SGPP’s recommendations were first published in 2025 (7). The SGPP’s recommendations were developed by two psychiatric experts in the field of coercive measures in psychiatry, who have several years of experience and have authored numerous publications on the subject. The full professors of psychiatry at the Universities of Geneva, Lausanne, Bern, Basel, and Zurich, as well as the executive boards of the SGPP and the Swiss Association of Chief Psychiatrists, contributed to the development of the recommendations. The final version of the recommendations was adopted and published by the SGPP executive board in 2025. The document comprises ten printed pages and is divided into four chapters: Introduction, prerequisites, prevention and conclusions. In terms of content, it offers pragmatic recommendations for action from a medical-psychiatric perspective. These recommendations are based on the AWMF S3-guideline “Prevention of Coercion: prevention and treatment of aggressive behavior in adults” from the German Society for Psychiatry, Psychotherapy and Neurology (8) as well as the SAMW guideline (6). The recommendations assume that the prevention of measures restricting liberty begins well in advance of the assessment of the prerequisites for ordering a commitment and thus often lies outside the sphere of influence of the psychiatric clinic. However, the rate of measures restricting movement and coercive treatments can be influenced by preventive measures within the clinic. Points of intervention include structural and procedural aspects as well as the staff attitudes and behavior. Concrete recommendations are provided in line with established guidelines and adapted to the Swiss context (7).

### 1.3 Position paper by the German-speaking section of the Swiss Society for Social Psychiatry on the topic of reducing coercive measures in psychiatry

The Swiss Society for Social Psychiatry (SOPSY) was founded in 1973. This interprofessional association aims to develop the conceptual framework of social psychiatry further, promote professional discourse and advocate within health policy for an approach to psychiatry that is community-based, participatory, and as free from coercion as possible (www.sozialpsychiatrie.ch). The position paper “Encounter instead of coercion: No Force First” was first published in 2021 and comprises two printed pages (9). The paper was developed by the Executive Board of the Swiss Society for Social Psychiatry, German-speaking Switzerland Section. It contains ten recommendations, including prioritizing relationship-oriented, dialogic and de-escalation approaches over measures that restrict freedom, ensuring adequate staffing levels in clinics, the systematic involvement of peers, the shift toward outpatient care, measures that promote autonomy such as crisis plans and advance directives, alternative approaches such as “open dialogue” and “Soteria”, as well as the complete abolition of restraints (9).

### 1.4 Charter for psychiatry without coercion – twelve theses with implementation examples from the Association of Psychiatric Nursing Managers Switzerland

The predecessor organization of the VPPS was founded in 1944. It is the central organization for leaders in psychiatric nursing in Switzerland, with a focus on professional development, quality, and leadership, as well as the interprofessional positioning of nursing (www.vpps.ch). The VPPS Charter was first published in 2025 and comprises eight printed pages (10). The working group consisted of representatives of the VPPS and the Academic Society for Psychiatric Nursing (Swiss Association for Nursing Science). They were supported by representatives of the family advocacy group “Stand by You Switzerland” and the EX-IN association. Following a preamble, the charter’s objectives are outlined, followed by twelve theses for psychiatry without coercion, each accompanied by three possible measures. The Charter for Psychiatry without Coercion views every measure that restricts freedom and every treatment administered without consent as fundamentally avoidable and calls for the consistent development of consensual, relationship-oriented alternatives on an equal footing. It also calls for dismantling of informal coercion and institutional power structures. To this end, it relies on specialized psychiatric nursing expertise, specific training, the incorporation of patients’ experiential knowledge, the active involvement of those affected and their relatives, as well as structural conditions that effectively render coercion unnecessary. At the same time, it calls for new, cross-institutional care models, close collaboration with referring agencies, the Child and Adult Protection Authority (KESB), the police, and policymakers, as well as a shared commitment to a psychiatry free of coercion at the leadership level across all professional groups (10).

### 1.5 Position paper by the Pro Mente Sana Foundation on coercive measures in psychiatry: “Strengthening Autonomy – Curbing Coercion”

The PMS Foundation was established in 1977. It is a non-governmental organization (NGO). Initially, the foundation was subsidized by the public sector (https://promentesana.ch/selbstbestimmt-genesen/klinikaufenthalt-und-selbstbestimmung/fuersorgerische-unterbringung). In return, the state entrusted the organization with various charitable tasks e.g. advancing the general interests of people living with mental disorders or disabilities. The organization engages in public outreach, political advocacy, and provides individual counselling and support. The position paper by PMS was drafted by the Foundation’s executive board and the Board of Trustees and adopted by the 2013 Foundation Assembly. It was first published in 2014 (11). The document comprises 10 printed pages and is divided into five chapters: Fundamentals, background, definition of coercive measures, demands of Pro Mente Sana, and areas of action for Pro Mente Sana, and focuses on patient rights and legal protection. The foundation calls for a consistent paradigm shift towards strictly human rights-based psychiatry, in which self-determination, informed consent and supported decision-making take precedence over paternalistic care. Coercive measures should be significantly reduced (by 50%) legally, institutionally, and culturally, recorded transparently, and systematically reviewed. PMS calls for independent complaint and oversight bodies as well as a strengthening of the procedural rights of those affected. Key areas for action include the expansion of outpatient, community-based, and peer-supported services, the promotion of crisis plans and advance directives, and prevention and consecutive de-escalation strategies in clinics. Additionally, PMS demands widespread, specialized training in professional conduct for specialists. Overall, these demands aim to establish structural frameworks that prevent the use of coercion, strengthen participation, and reduce power imbalances within the treatment system (11).

The aim of this review is to analyze the content of the five documents regarding the topics addressed and the resulting demands and recommendations and to identify both points of consensus and areas of controversy.

## 2 Methods

A narrative review was conducted with a particular focus on Swiss position papers and regulatory frameworks. Five documents published between 2014 and 2025 by different professional and stakeholder organizations were analyzed regarding their formal structure, key recommendations and discursive framing of coercion. For the purpose of comparison, coercion was understood broadly to include involuntary admission, treatment without consent, seclusion, restraint and forms of informal coercion. At the same time, the analysis considered that the documents differed in their implicit understanding of coercion, particularly regarding the distinction between restrictive safety measures and coercive treatment framed as therapeutic care under conditions of impaired decision-making capacity. The selection of documents was guided by a purposive sampling strategy aimed at capturing the core normative positions within the Swiss discourse on coercive measures in psychiatry. No additional statements and position papers exist that specifically addressed coercion in psychiatry in Switzerland. The selected documents can therefore be considered broadly representative of the dominant discursive positions, covering the most relevant professional, ethical and policy perspectives. Given the heterogeneity of sources, a structured narrative approach was employed to integrate normative and empirical perspectives, identify converging principles across position papers, and highlight existing gaps and inconsistencies in current practice. First, the main texts of the documents (PMS: Chapters 4 and 5; SAMW: Chapters 3 and 4; SOPSY: Page 2; SGPP: Chapter Prerequisites and Prevention; VPPS: 12 Theses) were reviewed and characterized based on their formal criteria (e.g., demands or recommendations) without considering introductions or definitions. Additionally, the thematic areas of the recommendations or demands regarding coercive measures in psychiatry were listed and presented in a comparative overview for all documents. Subsequently, the central points of consensus and controversies were identified and discussed in a thematic content analysis. Thematic areas were identified using an inductive-deductive approach by extracting and coding all recommendations and demands from the selected sections of the five documents and grouping them into higher-order categories. These categories form the basis of the comparative framework presented in **Table 1**, which maps the presence of each theme across documents. Agreement was defined as the occurrence of a theme in most or all documents with a similar normative orientation, whereas disagreement was assumed when themes were addressed differently in terms of scope, underlying assumptions or normative direction. The points of consensus and controversy reported in the Results section are therefore directly derived from the thematic distribution in **Table 1** and their qualitative interpretation in the respective texts. Levels of consensus were further differentiated by the frequency of thematic occurrence across documents: high consensus was defined as themes present in all or nearly all documents, moderate consensus as themes addressed by several but not the majority of documents, and low consensus or singular positions as themes appearing in only one or two documents. In addition, qualitative differences in emphasis and normative framing were considered by the authors to distinguish between mere thematic presence and substantive agreement. This approach allows for a more nuanced interpretation of consensus, beyond simple frequency counts. The five core papers were translated (German to English) using DeepL translator (2026).

**Table 1:** Subject areas of the recommendations / demands.

Topic	SAMW	SGPP	SOPSY	VPPS	PMS
Applies to involuntary commitment	✓	✓	✓	✓	✓
Applies to involuntary treatment	✓	✓	✓	✓	✓
Applies to restriction of movement	✓	✓	✓	✓	✓
Self-determination / Autonomy	✓	✓	✓	✓	✓
Conditions for coercion	✓	✓	–	–	✓
Incapacity (Involuntary committment)	–	✓	–	–	–
Incapacity (Involuntary treatment)	✓	✓	–	–	✓
Proportionality / Subsidiarity	✓	✓	–	–	–
Legal Framework (Implementation of the provisions of the Swiss Civil Code)	✓	✓	✓	–	✓
Advance Directive / Advance Planning	–	–	✓	✓	✓
Person of trust / Peers	✓	–	✓	✓	✓
Outpatient alternatives	–	✓	✓	✓	✓
Crisis intervention	–	✓	✓	✓	–
De-escalation / Communication	✓	✓	✓	✓	–
Human resources	–	✓	✓	✓	–
Attitudes / Values of professionals	✓	✓	✓	✓	✓
Reduction / Elimination of restraints	–	–	✓	✓	–
Informal coercion (e.g. persuasion, leverage oder threats)	✓	✓	–	✓	–
Structural / institutional factors	✓	✓	✓	✓	✓
Involvement of patients and their families	✓	–	✓	✓	✓
Research / Statistics	–	–	–	–	✓
Architecture / Building structures	–	✓	–	✓	–
Collaboration with police / authorities	–	✓	–	✓	–
Forensics	✓	–	–	–	–

## 3 Results

### 3.1 Summary of recommendations or demands

The SAMW publishes ethical and professional guidelines for medical practice that apply to the entire field of medicine, not just psychiatry. These guidelines contain four normative principles that are elaborated upon for six areas of application (somatic diseases, mental disorders, children and adolescents, long-term care, outpatient care, and the penal and correctional system): Respect for self-determination, subsidiarity and proportionality, appropriate environment, communication and documentation.

The SGPP’s recommendations offer a professional prevention strategy from a clinical-psychiatric perspective. The paper contains 19 recommendations regarding the implementation and compliance with legal requirements and the prevention of involuntary psychiatric admissions, treatment without consent, and measures restricting movement: A mental disorder requiring treatment as a prerequisite for involuntary psychiatric admission, risk to self or others, incapacity as a prerequisite for any measure restricting liberty, subsidiarity: explicitly consider alternatives, examine proportionality, ensure a suitable facility, preventive approach/attitude, “Zero Coercion” as a fundamental principle, Recovery-oriented approach, contextualization/needs-oriented approach, risk-neutral attitude, training in de-escalation and communication techniques, reflection on the use of informal coercion, diversified range of treatment and support services, psychiatric care in somatic emergency departments, differentiated psychosocial and psychiatric crisis intervention, cooperation with police/authorities, authority to issue restrictive orders, framework conditions in the clinic in accordance with the guidelines (8).

The SOPSY publication includes ten specific demands for social-psychiatric reform: Review staffing ratios, adequate resources for crisis situations, qualified staff in acute care units, systematic involvement of peers/trusted individuals, promote and fund outpatient care over inpatient care, promote crisis plans/advance directives, promotion of Open Dialogue/Soteria, trauma-minimizing implementation of involuntary treatment, coercive measures only for persons lacking capacity assessed by a treating psychiatrist who determines the need for coercion, abolition of restraints.

The VPPS publication presents a professional policy vision of psychiatry without coercion, comprising twelve theses, each with three examples of application: Relationships on an equal footing, avoiding/eliminating measures that restrict freedom, elimination of informal coercion, expertise in psychiatric nursing, education and training in alternative courses of action, utilizing experiential knowledge, involving those affected and their families in the development of treatment options, no delegation of crisis situations to the police or security services, facility designs that do not allow for measures restricting freedom, promote new care models, foster cross-system collaboration, a unified approach among leadership.

The PMS publication is a political-normative position paper containing ten specific demands and associated areas of action: Committal of persons lacking capacity by qualified physicians, incapacity regarding treatment without consent, binding nature of psychiatric advance directives, appointment of a trusted representative and establishment of independent services, conducting discharge interviews and drafting treatment agreements, restraint in the implementation of outpatient measures, promoting integrated, patient-centered care, promoting non-violent institutions, developing shared values within the team, improving statistics on involuntary admissions and expanding research.

None of the documents analyzed here are legally binding for individual psychiatric institutions. Deviations from recommendations or requirements do not require formal justification. The SAMW guidelines form part of the FMH (Swiss Medical Association) Code of Ethics and are recommended for use by the Swiss Association of Nurses.

**Table 1** presents the subject areas of the five documents in a comparative overview.

All five documents address the core domains of coercive practice in psychiatry, including involuntary admission, treatment without consent and measures restricting movement. Across documents, the term coercion is used with varying scope, ranging from narrowly defined restrictive measures to broader concepts including informal pressure, institutional power asymmetries and compulsory treatment. The overview shows that the topics of autonomy, the attitudes of healthcare professionals, and structural conditions appear in all five documents, while other topics - such as architecture and the avoidance of restraints- are only mentioned in some of the papers.

### 3.2 Points of consensus

There is a stable basic consensus, across all five papers, on the following main themes, whose recommendations and demands point in a common direction.

#### 3.2.1 Coercion as a last resort

All documents emphasize that coercive measures are permissible only as a last resort, must be subject to the principles of subsidiarity, proportionality, and time limitation, and require ethical justification on a case-by-case basis. Across all papers, there is consensus that voluntary treatment must always take precedence over measures restricting liberty, that such measures may only be used after milder alternatives have been exhausted, and that the total number of coercive measures should be minimized. The SAMW focuses on the implementation of legal-procedural aspects and ethical considerations. The SGPP recommends a strict interpretation and adherence to legal requirements as well as preventive approaches. SOPSY, PMS, and VPPS call for avoiding coercion whenever possible, refraining from physical restraints (SOPSY), specifically minimizing them (PMS, to 50% of the rate from the 2014 publication year), or eliminating them entirely (VPPS).

#### 3.2.2 Search for alternatives

All documents conclude that coercion can be potentially traumatizing for patients and staff, and therefore reflection and institutional learning are necessary following the use of coercive measures. All documents, except for SAMW, address the importance of treatment options alternative to inpatient care with low-threshold access for avoiding coercive measures, particularly outreach services and integrated treatment models.

#### 3.2.3 Prioritizing prevention

All documents agree that the most important strategy is not “proper implementation” but rather the avoidance of coercion. They emphasize, to varying degrees of explicitness and detail, that in direct contact with patients, de-escalation, relationship-building, and communication are the preferred means of avoiding coercion. As structural measures for the prevention of coercion, tools for advance planning for crisis situations are recommended. At the level of care organization, the needs-based expansion of outpatient, outreach, and community-based services is considered essential for the earliest possible and voluntary initiation of treatment and support. The topic of prevention is the focus of the SGPP’s recommendations for action and is presented here in a differentiated and detailed manner.

#### 3.2.4 Importance of attitude and relationship in prioritizing autonomy and self-determination

All documents emphasize the therapeutic relationship and a fundamental attitude that prioritizes the promotion and maintenance of autonomy to avoid coercive measures. It is emphasized that the quality of the relationship is central to preventing escalation and that institutional culture, team attitudes, and power asymmetries strongly influence the use of coercion.

Another point of consensus concerns the importance of societal, institutional, and organizational factors that influence coercion. Thus, staffing levels, staff qualifications, organizational culture, institutional structures, and regional care organization, as well as societal attitudes and willingness to include (SGPP), are significantly associated with the frequency and nature of the implementation of measures restricting freedom.

The involvement of affected individuals and their relatives in decision-making processes, particularly treatment planning, is included in the recommendations and demands of SOPSY, PMS, and VPPS.

The SAMW guidelines also address coercive measures in the context of criminal justice and the enforcement of measures. The other documents contain no explicit statements on the use of coercive measures in other contexts, particularly not in forensic settings.

In summary, it can be stated that there is consensus across all five documents that coercive measures constitute a serious infringement of fundamental rights and should be avoided as much as possible through psychiatric care that promotes autonomy, is preventive, and is structurally supported.

### 3.3 Controversies

Controversial points of discussion between the papers emerge in the following areas:

#### 3.3.1 ”Minimizing coercion” vs. “eliminating coercion”

SAMW, SGPP, and PMS accept that coercion is, in principle, unavoidable in exceptional cases, while SOPSY and VPPS advocate for a normative goal of “psychiatry without coercion.” SAMW and SGPP call for the minimization of measures that restrict freedom through legal and procedural rigor. SGPP extends this perspective to include a professionally effective preventive approach, noting the practical limitations imposed by a practice of institutionalization that is shaped by societal needs and attitudes and thus lies outside the direct sphere of influence of psychiatric professionals. PMS aligns closely with this position and calls for Switzerland to take a leading role in minimizing coercive measures by halving the number of such measures. SOPSY calls for the complete elimination of restraints and in line with the UN CRPD (12), advocates for the abolition of coercive measures (“No Force First”). VPPS posits that any measure restricting liberty is, in principle, avoidable and sets the goal of a psychiatry system entirely free of coercion. In this area of discussion, realistic pragmatism clashes with normative demands for transformation. The documents also differ implicitly in how they conceptualize different forms of coercion. While measures such as restraint or seclusion are predominantly framed as preventable escalations within institutional care processes, coercive treatment is in some documents partly justified as therapeutic intervention under conditions of impaired decision-making capacity, reflecting differing ethical interpretations of care, autonomy and protection.

#### 3.3.2 Role of capacity

In the Swiss context, the assessment of decision-making capacity is typically performed by the treating physician at the time of clinical evaluation and is closely linked to the decision on coercive measures. It is not generally conducted as a separate process by a court-appointed expert, although judicial review may follow in cases of involuntary admission. SAMW generally adheres to the existing legal framework and emphasizes a nuanced, legally precise conception of capacity. SGPP generally recommends establishing incapacity as a prerequisite for any measures restricting liberty, as such measures are not legitimate for persons with capacity (2). The PMS position paper contains nuanced explanations regarding the assessment of capacity and emphasizes that the presence of mental illness or a crisis should not lead to an assumption of incapacity without further examination. SOPSY calls for the legal restriction of coercive measures exclusively to persons lacking capacity. VPPS critiques the entire decision-making framework as an expression of power relations.

#### 3.3.3 The protective function of psychiatry

The question of psychiatry’s regulatory role (protective mandate, risk prevention, relationship with the police and authorities) is another point of controversy among the five documents. SAMW explicitly acknowledges that psychiatry has a protective function toward patients and third parties. It advocates for an ethically and legally balanced integration of autonomy, care, and harm prevention in psychiatry as a treatment system with a protective function. SGPP views the protective mandate, under strict conditions, as part of the psychiatric treatment system to avert acute self-harm directly associated with the illness. At the same time, it emphasizes that social control should not be the task of psychiatry. PMS acknowledges that psychiatry assumes a protective function to some extent but questions the overemphasis on the security discourse and highlights the danger of an inappropriate restriction of fundamental rights. SOPSY takes a significantly more critical stance toward the security discourse and calls for security to arise primarily through relationships, encounters, alternative services, and prevention. VPPS articulates the most radical position regarding the security discourse by stating that security-based reasoning can reinforce coercion and contradict a therapeutic approach. At the same time, it demands that individuals in crisis situations should not be handed over to the police or security services.

In summary, it can be stated that the lines of dissent among the listed “stakeholders” are multidimensional. The degree of need for reform, the objectives regarding coercive measures, the security function within psychiatry, and the associated interpretation of autonomy and the duty to protect represent key points of contention. Implicit assumptions make it difficult to consolidate positions based on reasoning.

## 4 Discussion

The current debate in Switzerland is, on the one hand, characterized by substantive contradictions and, on the other hand, influenced by the ongoing need for clarification regarding the increasingly frequent explicit demand that “psychiatry equals a secure space.” A clear stance by the medical profession on this matter will facilitate the contextualization of societal demands regarding “security.” Procedural frameworks ensure minimum legal and ethical standards but carry the risk of normalizing coercion as a “correctly implementable” practice. A recent European perspective emphasizes that safeguarding human rights during involuntary treatment depends less on formal legal criteria alone than on their consistent implementation through procedural safeguards, oversight mechanisms, and a rights-based clinical culture (13). In contrast, transformative-critical positions offer important impulses for reflecting on power, relationships, and institutional culture, but risk, if insufficiently differentiated, creating a decision-making vacuum and consequently, limited applicability in ongoing clinical practice.The SAMW’s medical ethics guidelines are intended for healthcare professionals and provide normative guidance on the use of coercive measures in practice. In terms of content, they emphasize the principles of self-determination, proportionality, and subsidiarity, and define conditions and procedures for the ethically justifiable use of coercion. It is the only one of the documents that considers the application of coercion on general somatic wards. In these wards most of the coercion is not monitored as rigorously as in psychiatric and mental health settings, and at the same time it is quite frequent (for example for patients who become delirious or aggressive and requiring urgent interventions). In these clinical situations, coercion is often done by medical reasons but could also be prevented or counteracted probably by the same principles that are described in psychiatric care. The SGPP’s clinical recommendations formulate approaches to preventing measures that restrict freedom from a specialist medical perspective, with a focus on legal requirements, structures, and attitudes within the legal framework. The SOPSY paper argues from a socio-psychiatric perspective on care and professional policy. It calls for structural reforms, better staffing resources, greater involvement of those affected, and the promotion of alternative treatment models to consistently reduce coercion. The VPPS Charter is a normative document on professional ethics that articulates a vision of “psychiatry without coercion.” In terms of content, it emphasizes a fundamental shift in attitudes, institutional structures, and care models in favor of a practice consistently oriented toward relationships and recovery. The PMS position paper is an advocacy-oriented document focused on patient rights, which aims politically and socially to reduce the use of coercive measures. In terms of content, it calls for a strengthening of self-determination, the expansion of patient-centered care, and legal and institutional reforms to minimize coercion.

The SAMW guidelines and the SGPP recommendations represent a professional-clinical perspective. Under the prevailing conditions, coercion is not fundamentally ruled out here but is accepted as a last resort when there are significant risks due to illness and milder measures have been exhausted. This position reflects the responsibility of psychiatric institutions for the treatment of severe mental health crises and the associated protection of the affected person and third parties. The documents from Pro Mente Sana and SOPSY take a more system-critical and reform-oriented perspective. They emphasize that the use of coercion is often linked to structural factors (e.g., lack of resources, institutional culture, lack of alternatives to inpatient treatment). Here, coercion is understood less as a medically necessary tool and more as an indicator of deficiencies in the care system. The VPPS Charter goes a step further and articulates a normative vision of psychiatry without coercion. While other documents accept coercion as a last resort, this document asks whether psychiatric practice could be organized fundamentally differently so that coercion becomes largely unnecessary.

These differing perspectives reflect central areas of tension in the discourse. A key area of tension exists between assigning a protective function to psychiatry (internal differentiation within psychiatry and demarcation from forensic psychiatry are absent here) in cases of severe self-harm or harm to others, and the right of those affected to self-determination and, consequently, to irrationality. Furthermore, there has recently been a growing body of research pointing to negative consequences following the use of coercion in psychiatric populations. A large Swedish cohort-study recently showed that individuals who received involuntary psychiatric care had a markedly increased risk of suicide, particularly during the first weeks after discharge (14). The study further showed that suicide risk was substantially higher compared with both voluntarily treated psychiatric patients and the general population, especially among younger men, individuals with personality or substance use disorders, and patients with a history of self-harm. Previously, a Swiss retrospective longitudinal study reported that coercive measures in psychiatric inpatient care were associated with significantly poorer mental health outcomes at discharge, providing robust evidence for a detrimental rather than therapeutic effect of coercion (15). Another area of tension concerns the question of to what extent coercive measures should be understood as the result of clinical decision-making processes in individual cases or as an expression of structural deficits in the care system. This finding is consistent with European data showing substantial variation in legal criteria and practices of compulsory admission across countries, suggesting that the use of coercion is shaped not only by clinical considerations but also by legal traditions and societal values (16). Finally, there is also a difference in normative objectives: while some documents aim primarily at reducing coercion, others articulate a more far-reaching vision of psychiatric care that, in the long term, functions without coercion.

The discourse on coercive measures must be conducted against the backdrop of societal expectations regarding risk minimization placed on law enforcement agencies (police, courts, government agencies, forensic services, etc.) and health institutions, medical and care facilities (psychiatric hospitals, residential homes and facilities, etc.). Societal expectations regarding a therapeutic and security-related mandate contribute significantly to the fact that, despite all critical evidence, coercive measures have thus far remained a legitimate tool for protecting the population. A central societal factor here is the widespread tendency to pathologize aggression and to view mental illnesses as potentially dangerous or uncontrollable (17). In public debates, amplified by media portrayals, violence is linked to mental illness, even though empirical studies show that the overall risk of serious violence by people with mental illness is low (18). Such interpretive patterns influence political and institutional debates. Pressure arises from a “no-risk” expectation to control risks as early as possible, which in turn influences the lowering of thresholds for the use of coercion. A societal shift toward a separation of powers, in which the “safe space” is not attributed to psychiatry as an institution, would require that mental illnesses be understood solely as complex health issues. Furthermore, societal attitudes toward self-determination play an important role. In many areas of medicine today, strong emphasis is placed on patient autonomy and participatory decision-making processes. In the context of mental health crises, however, there is a greater societal and professional willingness to accept paternalistic interventions, as demonstrated both in studies of public attitudes and in analyses of psychiatric treatment practices (19, 20). A reduction in coercion would require that the right to self-determination be more strongly recognized in psychiatry as well, even when decisions deviate from societal expectations. A reduction or abolition of coercive measures requires a societal willingness: tolerance toward uncertainty, as well as a shared responsibility to ensure support and care for people in mental health crises. This aligns with the WPA position statement, which emphasizes that reducing coercion requires not only respect for autonomy but also a societal commitment to developing accessible, community-based alternatives and shared systems of support for people in mental health crises (21).

Coercive measures in psychiatry are therefore a social issue in general population and should not be addressed solely within the medical field. A productive and goal-oriented discourse that also considers groups without a strong lobby and does not use humanistic ideas to forensicate medicine requires making existing differences in interests explicit and not hastily, through implicit communication, attempting to (pseudo-)harmonize them. It is crucial that the discussion does not remain confined to the professional community but is deliberately expanded into a broader societal dialogue. The goal is to address risk aversion in society with solidarity and not to disregard social shared responsibility for mental health crises. Reducing psychiatry to a “safe space” means ignoring decades of professional development. Interdisciplinary forums, political consultation processes, and joint evaluation or research projects can contribute to the development of evidence- and value-based strategies. At the same time, transparent and fair communication is essential to differentiate societal perceptions of mental illness, crises, and risks. The systematic inclusion of representatives from the media, those affected, and their families complicates the discourse in the short term but holds the potential for the development of solidarity-based and participatory reforms in the medium term. From the authors’ perspective, future progress in reducing coercion will depend less on further procedural refinement alone than on strengthening preventive, community-based and relationship-oriented models of care while maintaining clear legal safeguards.

## 5 Conclusion

- Coercive measures should be consistently framed as ultima ratio, with priority given to prevention, de-escalation and voluntary treatment across all settings.
- Effective reduction of coercion requires integration of procedural safeguards with relational, recovery-oriented and system-level interventions.
- Differences between documents highlight the need to explicitly address normative tensions, particularly regarding autonomy, protection, and the role of psychiatry in risk management.
- Structural factors such as staffing, institutional culture and availability of community-based alternatives are key determinants of coercion and must be systematically addressed.
- Future discourse should move towards shared societal responsibility, strengthening participation of service users and aligning legal, clinical and ethical frameworks to reduce coercion sustainably.
